# Hypoxia PET Imaging with [18F]-HX4—A Promising Next-Generation Tracer

**DOI:** 10.3390/cancers12051322

**Published:** 2020-05-22

**Authors:** Sebastian Sanduleanu, Alexander M.A. van der Wiel, Relinde I.Y. Lieverse, Damiënne Marcus, Abdalla Ibrahim, Sergey Primakov, Guangyao Wu, Jan Theys, Ala Yaromina, Ludwig J. Dubois, Philippe Lambin

**Affiliations:** 1 The D-Lab and The M-Lab, Department of Precision Medicine, GROW—School for Oncology, Maastricht University, 6211 Maastricht, The Netherlands; a.vanderwiel@maastrichtuniversity.nl (A.M.A.v.d.W.); relinde.lieverse@maastrichtuniversity.nl (R.I.Y.L.); d.marcus@maastrichtuniversity.nl (D.M.); a.ibrahim@maastrichtuniversity.nl (A.I.); s.primakov@maastrichtuniversity.nl (S.P.); g.wu@maastrichtuniversity.nl (G.W.); jan.theys@maastrichtuniversity.nl (J.T.); ala.yaromina@maastrichtuniversity.nl (A.Y.); ludwig.dubois@maastrichtuniversity.nl (L.J.D.); philippe.lambin@maastrichtuniversity.nl (P.L.); 2 Department of Radiology and Nuclear Medicine, GROW—School for Oncology and Developmental Biology, Maastricht University Medical Centre+, 6229 Maastricht, The Netherlands; 3 Division of Nuclear Medicine and Oncological Imaging, Department of Medical Physics, Hospital Center Universitaire De Liege, 4030 Liege, Belgium; 4 Department of Nuclear Medicine and Comprehensive Diagnostic Center Aachen (CDCA), University Hospital RWTH Aachen University, 52074 Aachen, Germany

**Keywords:** molecular imaging, tumor hypoxia, positron emission tomography (PET), [18F]-HX4, theranostics, response assessment

## Abstract

Hypoxia—a common feature of the majority of solid tumors—is a negative prognostic factor, as it is associated with invasion, metastasis and therapy resistance. To date, a variety of methods are available for the assessment of tumor hypoxia, including the use of positron emission tomography (PET). A plethora of hypoxia PET tracers, each with its own strengths and limitations, has been developed and successfully validated, thereby providing useful prognostic or predictive information. The current review focusses on [18F]-HX4, a promising next-generation hypoxia PET tracer. After a brief history of its development, we discuss and compare its characteristics with other hypoxia PET tracers and provide an update on its progression into the clinic. Lastly, we address the potential applications of assessing tumor hypoxia using [18F]-HX4, with a focus on improving patient-tailored therapies.

## 1. Introduction

Hypoxia is a common feature of the majority of solid tumors and arises due to a disturbed balance between proliferation and oxygen supply [1]. Tumor hypoxia contributes to resistance to radio- and chemotherapy, invasion and metastasis [2] and is associated with more aggressive cancer phenotypes and poor prognosis. Methods to accurately and reproducibly detect and quantify tumor hypoxia can hence improve patient outcome by not only serving as a prognostic factor, but also by allowing selection of more appropriate therapies—i.e., providing predictive information—to overcome tumor hypoxia and its effects.

To date, a variety of methods are available for assessing tumor hypoxia, including the use of the oxygen electrodes and immunohistochemical assays [3]. Despite the use of these gold-standard invasive modalities, it remains difficult to measure oxygen levels reproducibly in a highly heterogeneous three-dimensional (3D) tumor environment [4]. Research has therefore been focused on the development of noninvasive techniques that provide spatially resolved quantitative images. Even though imaging of endogenous markers of hypoxia, such as carbonic anhydrase IX (CAIX) and vascular endothelial growth factor (VEGF), has been employed for this purpose, hypoxia-specific positron-emission tomography (PET) radiotracers have the advantage of directly reflecting tumor oxygen levels rather than hypoxia-mediated changes in phenotype [5].

**Figure 1:**
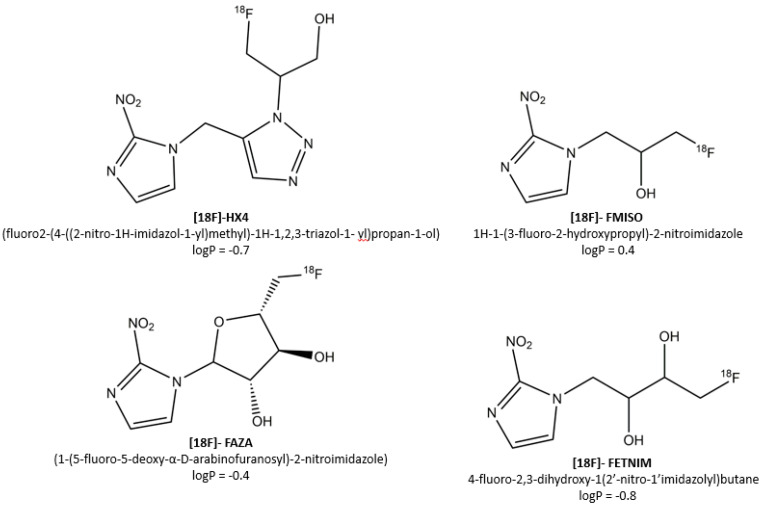
The currently most-investigated 2-nitroimidazole PET tracers and their associated logP values.

Given the clinical relevance of tumor hypoxia and the increasing need for patient-tailored therapies, a large number of hypoxia PET tracers have been developed and evaluated. More recently, the third-generation 2-nitroimidazole nucleoside analogue [18F]-flortanidazole ([18F]-HX4) was developed and validated (Figure 1) [6], which demonstrated highly promising preclinical and clinical results. This review summarizes the development of [18F]-HX4, its benefits and limitations, and provides an update on its current clinical application.

## 2. Characteristics of an Ideal Hypoxia PET Marker

Since the introduction of misonidazole (MISO) in 1981 as the first marker for molecular imaging of tumor hypoxia [7], a plethora of hypoxia PET tracers has emerged. Each of these tracers must ideally possess a number of distinct characteristics [5], both from a PET imaging perspective and a clinical point of view. First, the PET tracers should be able to identify regions with oxygen levels within the clinically relevant range, regardless of tumor type or stage. This means that both severely hypoxic regions (<0.5mmHg O_2_) and regions with intermediate levels of hypoxia (0.5–10mmHg O_2_) should be detected, as the latter regions can be more important in determining the tumoral response to, e.g., fractionated radiotherapy [8,9]. Second, its pharmacokinetic profile should allow a homogenous distribution—regardless of factors such as blood flow or pH that can co-vary with hypoxia—and rapid elimination from normoxic tissues. The primary determinant of the pharmacokinetics of a tracer is the octanol-water partition coefficient—and its logarithm (logP)—indicating the hydro- or lipophilicity of a compound. In general, more lipophilic tracers are characterized by rapid distribution and tumor uptake at the expense of impaired background clearance [10], whereas the opposite holds true for more hydrophilic markers. Lastly, from a clinical point of view, an ideal PET tracer should be easy to synthesize, and should allow for accurate, repeated measurements within short post-injection (PI) acquisition times.

The clinical utility of a tracer is thus ultimately determined by all of the characteristics described above, as they influence its hypoxic specificity, the timeframe within which imaging must be performed (i.e., the optimal acquisition time), and its availability for clinical use. Table 1 summarizes the four most widely investigated 2-nitroimidazole tracers and how these agents compare to the characteristics of the ideal hypoxia PET tracer [5], as previously well-defined by Fleming et al. [11].

## 3. The Development of ^18^F-HX4: A Promising Third-Generation Hypoxia PET Tracer

Despite extensive research on a wide range of hypoxia PET tracers as already thoroughly reviewed by others [11,31], not a single of these tracers is currently European Medicines Agency (EMA) and the United States (US) Food and Drug Administration (FDA) approved, putatively due to lack of robust multicentric imaging studies and concerns regarding cost-effectiveness in routine clinical setting [32]. Up to date, the fluorinated nitroimidazole derivative [18F]-fluoromisonidazole (1H-1-(3-fluoro-2-hydroxypropyl)-2-nitroimidazole, [18F]-FMISO) remains the most extensively studied tracer—both preclinically and clinically—for PET imaging since its development in 1986 [33] and first validation in patients in 1992 [34]. Notwithstanding this, the clinical utility of [18F]-FMISO is limited as only modest signal-to-noise ratios—and therefore images with only moderate contrast—are obtained due to its high relatively lipophilicity and slow clearance [21].

These limitations of [18F]-FMISO have led to the development of second-generation, more water-soluble 2-nitroimidazole analogues with improved pharmacokinetic properties to enhance signal-to-noise ratios. Preclinical validation of the second-generation 2-nitroimidazoles [18F]-FAZA (1-(5-fluoro-5-deoxy-α-D-arabinofuranosyl)-2-nitroimidazole) and [18F]-FETNIM (4-fluoro-2,3-dihydroxy-1(2’-nitro-1’imidazolyl)butane) indeed showed improved tumor-to-background ratios when compared to [18F]-FMISO [35,36]. In line with this, Souvatzoglou et al. concluded in a study in 11 head and neck squamous cell carcinoma (HNSCC) patients that PET imaging with [18F]-FAZA is slightly more favorable when compared to [18F]-FMISO at earlier time points [37]. [18F]-FETNIM, in contrast, showed lower uptake and tumor-to-blood ratios than [18F]-FMISO in a more recent study in 42 lung cancer patients [38]. In addition to these more water-soluble hypoxia PET tracers, more lipophilic fluorinated compounds such as EF3 have also been investigated as promising alternatives. However, in a comparative preclinical rat model, EF3 failed to show superiority to [18F]-FMISO for the evaluation of hypoxia [39]. Lastly, Cu-labeled diacetyl-bis (N^4^-methylthiosemicarbazone) analogues (Cu-ATSM) are a different class of hypoxia PET tracers demonstrating retention under hypoxic conditions [40]. Nevertheless, its specificity for hypoxia has shown to be potentially dependent on tumor type [41], and the relationship with Cu-ATSM uptake and tumor oxygenation status has proven to be complex [24], making these compounds—at least without further research—less promising candidates for assessment of tumor hypoxia in clinical setting.

**Figure 2:**
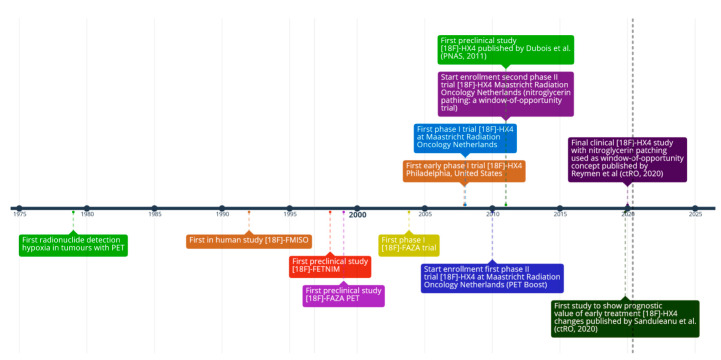
History of hypoxia PET, with a focus on the third generation PET tracer ^18^F-HX4.

More recently, the third-generation 2-nitroimidazole nucleoside analogue [18F]-flortanidazole ([18F]-HX4) was developed (Figure 2) [6]. By using synthetic convenient click chemistry [42], a 1,2,3-triazole moiety was incorporated, rendering the compound more hydrophilic (logP = −0.69) when compared to [18F]-FMISO (logP = −0.40) and [18F]-FAZA (logP = −0.4) [6,43,44]. In addition to increased hydrophilicity, the 1,2,3-triazole moiety further improved clearance of ^18^F-HX4 by promoting renal clearance [45]. These characteristics of [18F]-HX4 will hence contribute to a faster decrease of background signal when compared to tracers with a slower plasma half-life such as [18F]-FMISO, leading to improved signal-to-noise ratio [6]. The downside of the enhanced renal clearance of [18F]-HX4 is that bladder voiding has to be ensured in order to prevent excessively high bladder wall dosimetry.

**Figure 3:**
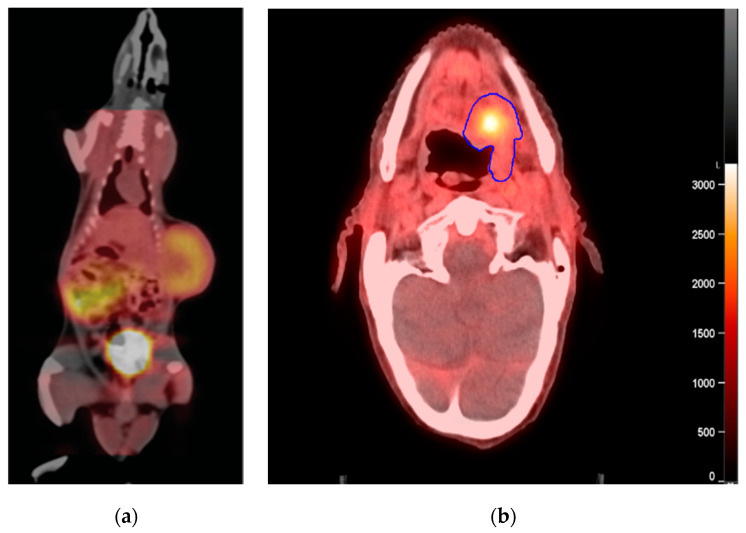
HX4 tracer accumulation in rats and patients. Units are provided in Becquerel/milliliter (Bq/mL) (**a**) Representative fused CT and PET image of a rhabdomyosarcoma R1 tumor-bearing rats 4 h after injection of [18F]-HX4. In this rat, tracer accumulation can be observed in the tumor and bladder, indicating selective tumor retention and renal excretion, respectively. Reproduced from Dubois et al., PNAS [6] (**b**) Representative fused CT and PET image of a head and neck cancer patient (NCT01504815 trial) 4 h after injection of [18F]-HX4 with defined primary gross tumor volume (blue) contour.

Upon synthesis of HX4 by means of the click chemistry approach, one crucial step was the validation by Dubois et al. of a clear causal relationship between [18F]-HX4 uptake and tumor oxygenation in a preclinical rat rhabdomyosarcoma tumor model employing modified breathing strategies (Figure 3) [6]. Furthermore, a strong and significant spatial relationship was observed between [18F]-HX4 distribution and pimonidazole [6,45,46] and carbonic anhydrase 9 (CAIX) positivity [45,46], indicating that [18F]-HX4 specifically accumulates in hypoxic regions [6,45]. Unfortunately, there are no published studies to date comparing [18F]-HX4 uptake and tumor oxygenation based on 3D pimonidazole positivity of the tumor, though the currently accepted gold standard for assessing tumor hypoxia is from pimonidazole staining from at least five places in the tumor [13].

[18F]-HX4 has also been evaluated in several clinical trials (Table 2). In a Phase I trial in patients with stage 4 non-small cell lung cancer (NSCLC), [18F]-HX4 was well tolerated without any observed toxicities, and a good correlation between hypoxic areas indicated by [18F]-HX4 and areas of high [18F]-FDG uptake was observed in three out of six patients in whom an [18F]-FDG was performed [47]. A similar correlation was later found in HNSCC patients on a global tumor level; however, a partial mismatch between [18F]-FDG and [18F]-HX4 uptake emphasized that [18F]-FDG PET cannot be used as a surrogate for assessment of tumor hypoxia [4]. Similar studies deemed the assessment of tumor hypoxia using [18F]-HX4 PET feasible and favorable in NSCLC [48], head and neck squamous cell carcinoma (HNSCC) [49,50], esophageal and pancreatic cancer [30]. Importantly, the amount and localization of [18F]-HX4 PET demonstrated good repeatability [4,30], underscoring its potential use as a tool for, e.g., treatment response monitoring and radiation therapy planning (*vide infra*).

## 4. Comparison of [18F]-HX4 to Other Hypoxia PET Tracers

Despite the clear clinical utility of hypoxia PET tracers, it remains difficult to qualitatively and quantitatively compare one to the other currently available tracers due to a lack of standardized image acquisition and analysis (e.g., acquisition time, choice of background tissue, a threshold to define hypoxia), and a large variety in tumor models or cancer types studied. In addition, few and typically small comparative studies have been performed between hypoxia PET-tracers and multiparametric imaging to assess tumor metabolism and vasculature [51].

In a preclinical rat rhabdomyosarcoma model, Peeters et al. assessed and compared crucial characteristics of the PET tracers [18F]-FMISO, [18F]-FAZA, and [18F]-HX4 [52]. Differences in tumor uptake of the tracers resulted in a significantly higher maximum tumor-to-blood ratio for [18F]-HX4 when compared to those of [18F]-FMISO and [18F]-FAZA. Furthermore, as expected given their high hydrophilicity, clearance of [18F]-FAZA and [18F]-HX4 was markedly increased when compared to that of [18F]-FMISO. In line with this, the optimal acquisition time for [18F]-FAZA (2 h p.i.) and [18F]-HX4 (3 h p.i.) was reached earlier; [18F]-FMISO did not show plateau formation and tumor-to-blood ratios continued to increase up to 6 h p.i., as clinically demonstrated. These findings were also reflected in the higher biological half-life in normal tissues of [18F]-FMISO (4.5 h p.i.) than [18F]-HX4 (2.2 h p.i.) and [18F]-FAZA (2.8 h p.i.). Lastly, when comparing consecutive scans taken 48 h apart, only [18F]-FMISO and [18F]-HX4 demonstrated good reproducibility. A different preclinical study comparing the same three PET tracers found similar tumor-to-muscle ratios for [18F]-FMISO, [18F]-FAZA, and [18F]-HX4 90 min p.i [46], even though [18F]-FAZA demonstrated reduced absolute tumor and normal tissue uptake. Nevertheless, it should be noted that the time point of evaluation (i.e., 90 min p.i.) might be too early, as normal tissue clearance is still ongoing.

There is only one clinical [18F]-HX4 study so far testing two different tracers in the same patient population. By comparing ^18^F-FMISO with [18F]-HX4 in 12 HNSCC patients, Chen et al. demonstrated similar tumor-to-muscle ratios for [18F]-HX4 and [18F]-FMISO images acquired, respectively, 1.5 and 2 h p.i. [49], suggesting a potential advantage of [18F]-HX4 of a shorter acquisition time. Nevertheless, it should be noted that both for [18F]-FMISO and [18F]-HX4, image acquisition at later time points can enhance signal-to-noise ratios, resulting in a more accurate assessment of tumor hypoxia. This was demonstrated in a study by Zegers et al., which showed that the highest tumor-to-blood ratio for [18F]-HX4 was achieved 4 h p.i. [53]. More recently, a mathematical simulation performed by Wack et al. estimated that—despite having the lowest absolute tracer activity four hours post-injection and highest interpatient variation—[18F]-HX4 demonstrated the highest simulated contrast (2.31 vs. 1.67 h ([18F]-FMISO) and 1.75 ([18F]-FAZA) [54].

It becomes evident that the ultimate hypoxia tracer does not exist, and that each tracer is characterized by its own strengths and weaknesses that can be exploited for the specific (research) question to be answered. In addition, a factor not to be forgotten in choosing the most suitable PET tracer are the costs associated with its use. When comparing the costs of [18F]-HX4 to the other tracers, these can vary from country to country and depend not only on the costs of radiotracer synthesis, but likewise on quality assurance, costs of scanner depreciation, and transportation of the radiotracer across centers. In the UK, the total hypoxia PET-scan costs can amount to £2000–£3000/scan [55]. In the radiosynthesis process, the costs of the radiolabeling process by means of click chemistry are lower compared to the production costs of the radionuclide [55,56].

## 5. Clinical Applications of [18F]-HX4 Hypoxia PET

Given the prevalence of tumor hypoxia and its therapeutic importance, it is clear that hypoxia PET imaging has multiple applications. Several hypoxia PET tracers, including [18F]-HX4, have shown to provide useful prognostic and/or predictive information and allow monitoring of treatment response, most notably in the case of dose painting studies based on hypoxia. Furthermore, we will discuss the potential roles of hypoxia PET in newer hypoxia modifying therapies, both under active clinical investigation (e.g., hypoxia activated pro-drugs) as well as in vitro.

## 6. Prognostic Value of Hypoxia PET Imaging [18F]-HX4 PET

One of the important objectives of assessing tumor hypoxia using PET is the identification of patients with a poor prognosis, and, consequently, which individuals are more likely to benefit from therapies focusing on overcoming tumor hypoxia. Indeed, it has already been demonstrated on several occasions and in multiple cancer types that a high uptake of the PET tracers [18F]-FMISO [57,58,59,60], [18F]-FAZA [61], and [18F]-FETNIM [60,62] is a predictor of poor treatment response and prognosis. In further support of this, a large meta-analysis on published hypoxia PET studies demonstrated that patients with more hypoxic tumors responded significantly poorer to radiotherapy [63].

Evidence regarding the prognostic value of [18F]-HX4 uptake, on the other hand, is less extensive. In a preclinical breast cancer model, Yu et al. found that a higher [18F]-HX4 uptake at baseline was associated with a worse prognosis, regardless of treatment [64]. Furthermore, tumoral baseline [18F]-HX4 uptake was positively correlated with tumor growth rate in a lung [65] and colorectal cancer xenograft model [66]. Only recently, Reymen et al. were the first to report in a study of 42 NSCLC patients that the degree of tumoral [18F]-HX4 uptake was negatively correlated with prognosis [20].

## 7. Monitoring Treatment Response [18F]-HX4 PET

The ability to monitor the treatment response can provide valuable information, allowing treatment modification in an early stage. It has already been demonstrated that, by using the hypoxia PET marker [18F]-FMISO and the metabolic marker [18F]-FDG, early treatment-induced changes could be detected and, interestingly, that these changes showed stronger association with treatment outcome that the pretreatment measurements [67,68].

As stated already above, the amount and localization of [18F]-HX4 PET demonstrated good repeatability between scans, underscoring its potential use as a tool for monitoring and prediction of treatment response. This was further by Yu et al., who demonstrated a clear reduction in hypoxic volume assessed by [18F]-HX4 uptake after radiotherapy treatment in a preclinical human breast cancer xenograft model [64]. In addition, they found that a higher [18F]-HX4 uptake at baseline was associated with a worse prognosis. In line with these findings, [18F]-HX4 PET has been successfully used to determine treatment response to metformin, an antidiabetic drug with anticancer properties, in animal models of NSCLC and colorectal cancer [66]. In clinical setting, Reymen et al. [20] assessed treatment response to nitroglycerin as a potential radiosensitizer using [18F]-HX4 in patients suffering from NSCLC. They found that administration of nitroglycerin did not result in the hypothesized decrease of tumor hypoxia defined as [18F]-HX4 uptake, more indicative of the arguable potential of nitroglycerin as a radiosensitizer.

Furthermore, in HNSCC patients, Zegers et al. demonstrated decreasing tumor hypoxia during the course of treatment with (chemo) radiotherapy [50]. Interestingly, patients with a high baseline uptake of [18F]-HX4 were most likely to exhibit persistent hypoxia during treatment. Unfortunately, as accrual was still ongoing, the correlation between change in tumor hypoxia during treatment and the actual treatment outcome could not be assessed. Nevertheless and despite limited evidence, these results suggest that [18F]-HX4 PET can be used to monitor treatment response [20].

## 8. Selection for Hypoxia-Targeted Therapies

Even though hypoxia is one of the best-validated targets in oncology, its full potential still remains to be exploited in the clinical setting. Nevertheless, several strategies to combat tumor hypoxia have been proposed and explored to improve therapy outcome, including the use of hypoxia-activated prodrugs (HAPs), which exert their cytotoxic effects only in hypoxic tumor regions. Surprisingly, over 50 years of HAP design has failed to deliver a clinically approved agent as the majority of HAP clinical trials yielded disappointing results. For example, despite highly promising Phase II clinical trials [69], two recent phase III trials failed to demonstrate the effectivity of TH-302 in increasing overall survival in patients suffering from pancreatic cancer (NCT01746979) [40] and soft tissue sarcoma (NCT01440088) [70], which resulted in the withdrawal of TH-302. Potential reasons explaining this failure include a lack of patient stratification based on tumor oxygenation status. Hence, assessing the degree of tumor hypoxia using PET imaging might provide a powerful tool for patient selection and—ultimately—successful clinical translation of HAPs [71].

In this context, a post-trial retrospective analysis Rischin et al. demonstrated that the addition of the HAP tirapazamine was only beneficial in patients with tumor hypoxia as indicated by ^18^F-FMISO uptake [69]. In patients with no detectable tumor hypoxia, additional treatment with tirapazamine was not effective, supportive of patient stratification based on hypoxia PET. However, it should be noted that in this post-trial retrospective analysis, tumor hypoxia using [18F]-FMISO was not assessed in all patients. The HAP PR-104 also has been investigated in a clinical setting in combination with [18F]-FMISO (NCT00862134); however, no correlation between baseline [18F]-FMISO uptake and antitumor effects of PR-104 was found [72]. For [18F]-HX4, similar findings have been reported albeit in a preclinical model, where an association was observed between pretreatment tumor hypoxia as assessed by [18F]-HX4 tumor uptake and therapeutic efficacy of the HAP TH-302 in combination with radiotherapy [73]. Due to the withdrawal of TH-302 as described above (*vide supra*), a Phase I window-of-opportunity trial (patients receiving one or more novel compounds such as HAPs between their diagnosis and standard-of-care) exploring the relationship of [18F]-HX4 uptake at baseline and efficacy of TH-302 in esophageal cancer patients was discontinued (NCT02598687) [74]. Up to date, tirapazamine and PR-104 remain the only HAPs investigated in combination with hypoxia PET imaging in a clinical setting. Besides HAPs, other hypoxia-targeting therapies are likely to benefit from patient stratification based on hypoxia PET, e.g., by decreasing cellular oxygen consumption and improving tumor oxygenation [9]. Nitroglycerin, a vasodilating agent, for example, has been investigated as a proposed tumor hypoxia modifier. However, a recent clinical trial (NCT01210378) failed to demonstrate reduced tumor hypoxia as assessed by [18F]-HX4 PET upon nitroglycerin administration [20].

Taken together, several hypoxia-targeted therapies have been developed and evaluated in the clinic, often with disappointing results. We believe and emphasize that the use of hypoxia assessment and subsequent patient stratification is of utmost importance for the success of these hypoxia-targeted therapies and future personalized cancer medicine.

## 9. Patient Stratification for FLASH Therapy

Tumor hypoxia increases resistance to radiotherapy and systemic therapy. The FLASH effect, irradiation at ultra-high dose rate (>40 Gy/s) with an extremely short irradiation time, is hypothesized to deplete oxygen too quickly for diffusion to maintain an adequate level of oxygenation, and consequently, the normal tissue will respond as a hypoxic tissue [75]. When a hypoxic tumor is surrounded by normoxic tissue, the ultra-high dose rate will increase the radioresistance of the normal tissue with small impact on the already hypoxic tumor tissue.

Animal models have in a few cases shown good skin-sparing and tumor response equivalent to standard regimens with FLASH radiotherapy [75]. The results of a recent in vitro study suggest that when compared to conventional radiotherapy, FLASH minimizes the DNA damage in normal cells, spares lung progenitor cells from excessive damage and reduces the risk of replicative senescence [76].

Biomarkers based on hypoxia PET-tracers as well as newer tracers such as HX4 could be used to assess hypoxia status in vivo.

## 10. Patient Stratification for Systemic and Radiation Therapy

Stratifying patients undergoing ARCON (accelerated radiotherapy with carbogen and nicotinamide) based on their pre-therapeutic hypoxic status (pimonidazole staining) demonstrated that the benefit in loco-regional control was specifically observed for patients with initial tumor hypoxia before the start of treatment [77,78].

In an attempt to assess early changes in hypoxia Lock et al. evaluated re-oxygenation during the course of treatment with [18F]-FMISO PET in an exploratory cohort and a validation cohort, both consisting of 25 patients [79]. Instead of fixed cutoff value, the pre-treatment [18F]-FMISO-PET of each individual patient served as intra-patient control to calculate the residual hypoxia volume at week 1, 2, and 5. A significant decrease in loco-regional control for tumors with residual hypoxia could be shown in the exploratory cohort and successfully validated in the validation cohort; the strongest predictive value was found in the second week of treatment. Mortensen et al. used [18F]-FAZA PET at baseline in a cohort of 40 patients with HNSCC to show significantly improved disease-free survival in non-hypoxic tumors compared to hypoxic tumors [60]. Only 13 patients had a second FAZA PET during treatment after a median 14 days of which most had no residual hypoxic volume. Treatment failure occurred in four out of six patients with residual hypoxic volume compared to two out of seven patients with no residual hypoxia.

With regard to the possibility for dose painting based on [18F]-HX4 PET, Sanduleanu et al. [80] and Busk et al. [28] have found that there is great variability in the PET-signal (low voxel-wise PET correlation coefficients) between two timepoints early during the course of treatment and the same PET-scan at different post-injection intervals. These findings pose a substantial technical challenge for upcoming dose painting studies. One of the proposed methods for dose escalation in a preclinical study [80] showed promising results. In this study, inverse radiation dose-painting was performed to boost selectively non-hypoxic tumor sub-volumes having no/low hypoxia-activated pro-drug uptake.

Withthe recent development of a several biomarkers and PET tracers to assess tumor hypoxia, from gene profiles to radiomics imaging biomarkers, there is someevidence that these can be used as theranostic markers [81,82,83].

We believe that the use of hypoxia PEThas the potential to be part of routine patient care, granting valuable predictive or prognostic information ultimately improving patient outcome.

## 11. Conclusions

Hypoxia is a common feature of most solid tumors and is associated with poor prognosis and resistance to conventional therapies. To date, several methods are available for the assessment of tumor hypoxia, including the use of hypoxia PET tracers. These tracers not only showed to be reliable for the assessment of tumor hypoxia, they also demonstrated their prognostic significance, potential to predict and monitor treatment response, and improve personalized cancer medicine. Even though the ideal hypoxia PET tracer does not exist, [18F]-HX4 is a promising next-generation tracer with several favorable properties. We believe that hypoxia PET tracers are a promising tool—if their limitations are successfully overcome—to provide valuable predictive and prognostic information, to train radiomics signatures and to support the development of hypoxia targeting therapies, thereby ultimately improving patient outcome.

## Figures and Tables

**Table 1:** Summary of the four currently most used and tested 2-nitroimidazole hypoxia positron-emission tomography (PET) tracers ([18F]-FMISO, [18F]-FAZA, [18F]-FETNIM, [18F]-HX4), and how these compare to the characteristics of the ideal hypoxia PET tracer.

	Characteristic	Tracer			
		[18F]-FMISO	[18F]-FAZA	[18F]-FETNIM	[18F]-HX4
1	Hypoxia specificity	[5,12,13]	[5]	[5]	[5,6]
2	Well-defined mechanism of retention	[14]	[15]	[16]	[4]
3	Homogenous distribution and rapid clearance	[5]	[5]	[5]	[4,5,6]
4	Little dependency on factors that co-vary with hypoxia	[17]	[18]	[19]	[20]
5	Stability against non-hypoxic metabolism	[6,21]	x	x	[6]
6	Suitable acquisition time	[22]	[23]	[23]	[23]
7	Easy to synthesize and readily available	[24]	[24]	[24]	[24,25]
8	Amenable dosimetry profile	[5,26]	[5]	[26]	[26]
9	Repeatability spatial uptake	[27]	[28]	[29]	[30]
10	Effective regardless of tumor type and stage	[5]	[5]	[5]	[5]

Green: characteristic met; yellow: no consensus; red: characteristics not met; gray: no data available. Definition of characteristics: (1) the tracer should be retained in regions with hypoxia within the clinically relevant range; (2) the mechanism of cellular retention should be well-defined and independent of cell type; (3) the tracer should be sufficiently lipophilic to enter cells and allow uniform tissue distribution, but also sufficiently hydrophilic to avoid membrane sequestration, and have faster clearance from systemic circulation and normoxic tissue; (4) its pharmacokinetic profile and tissue distribution should exhibit little dependence on parameters that may co-vary with hypoxia, such as blood flow or pH; (5) it should have high stability against non-hypoxia specific metabolism in vivo; (6) its tissue kinetics should be suitable for imaging within a timeframe permitted in the clinical setting; (7) it should be easy to synthesize and readily available; (8) it should possess a favorable radiation dosimetry profile; (9) it should be repeatable to allow both detection of hypoxia and return to normoxia; (10) it should be effective in multiple tumor types and stages.

**Table 2:** Overview of all active, completed, or terminated clinical trials up to date using [18F]-HX4 for the detection of tumor hypoxia.

Name	Phase	Type	Status	Cancer Type	Patients	Aims and Results	Sponsor	Associated Publications	Study Identifier
**DHX4000**	I	Monocentric (USA)	Completed	HNSCC	4	Assessment of safety and biodistribution of [18F]-HX4. 80% of [18F]-HX4 maintained its integrity 2 h p.i. and cleared quickly through the renal system. High-quality PET images can be obtained shortly p.i.	Siemens Molecular Imaging (Siemens Healthineers AG, USA)	[44]	NCT00606424
**08-3-040**	I	Monocentric (EU)	Completed	Lung; Colon	6	Determination of the toxicity of [18F]-HX4. No signs of toxicity were observed.	Maastricht Radiation Oncology (NL)	[46]	NCT00690053
**PET Boost**	II	International multicentric (EU/UK)	Active, not recruiting	NSCLC	150	[18F]-FDG PET-based irradiation boost. [18F]-HX4 PET was included. An overlap between hypoxic and metabolically active volumes was observed.	Netherlands Cancer Institute (NKI-AVL, NL)	[50]	NCT01024829
**HX4-200**	II	National multicentric (USA)	Completed	HNSCC; Lung; Liver; Rectal; Cervical	50	Assessment of the reliability of [18F]-HX4 PET. A high association between first and second HX4 PET, based on SUV_max_ (R = 0.883), SUV_mean_ (R = 0.887), and TBR (R = 0.945) was found.	Siemens Molecular Imaging (Siemens Healthineers, USA)	N/A	NCT01075399
**Nitroglycerin in NSCLC**	II	Monocentric (EU)	Terminated (after futility analysis)	NSCLC	47	Assessment of the potential of nitroglycerin as radiosensitizer, and whether [18F]-HX4 can be used for patient selection.	Maastricht Radiation Oncology (NL)	[50]	NCT01210378
**HX4/FMISO**	II	Monocentric (CN)	Completed	HNSCC; Lung; liver	12	Evaluation of [18F]-HX4, and comparison to [18F]-FMISO. [18F]-HX4 has higher sensitivity and specificity, faster clearance, and shorter acquisition time p.i. compared with [18F]-FMISO	PET center, Huashan Hospital, Fudan (CN)	[48]	NCT01213030
**2011-001812-80**	II	Monocentric (EU)	Completed	HNSCC	23	Assessment of imaging parameters of [18F]-HX4 PET and correlation with [18F]-FDG uptake. A correlation between [18F]-HX4 and [18F]-FDG uptake was found, even though a partial mismatch was observed.	Maastricht Radiation Oncology (NL)	[49]	NCT01347281
**MIPA**	II	National multicentric (EU)	Completed	Pancreas	47	Assess whether [18F]-HX4 can be used as a tool to predict treatment outcome in pancreatic cancer. (#)	University of Amsterdam (UMC-UvA, NL)	N/A	NCT01989000
**HYPE**	II	Monocentric (EU)	Completed	Esophageal; Pancreatic; Rectal	32	Assessment of the optimal imaging parameters and reproducibility of [18F]-HX4 PET. Optimal acquisition time was found to be 3–4 h p.i., with good repeatability between different acquisitions.	University of Amsterdam (UMC-UvA, NL)	[30]	NCT01995084
**HX4-cervix**	II	Monocentric (EU)	Terminated (patients did not want to participate)	Cervix	4	Assessing tumor hypoxia using [18F]-HX4, investigate optimal acquisition time, and compere [18F]-HX4 uptake with [18F]-FDG uptake and blood and tissue markers.	Maastricht Radiation Oncology (NL)	N/A	NCT02233387
**CHLOROBRAINII**	II	Monocentric (EU)	Active, not recruiting	GBM	156 *	Assessment of the added value of chloroquine on treatment for GBM. Tumor hypoxia will be assessed using [18F]-HX4 PET. (#)	Maastricht Radiation Oncology (NL)	N/A	NCT02432417
**HX4 SD**	II	Monocentric (EU)	Terminated (patients did not want to participate)	Prostate; Esophageal; Brain primary; Brain metastases; Rectal	1	Assessing and visualizing tumor hypoxia using [18F]-HX4 and exploring the relationship between [18F]-HX4 uptake and tumor recurrence and survival.	Maastricht Radiation Oncology (NL)	N/A	NCT02584400
**OXYPET**	II	Monocentric (UK)	Completed	HNSCC; NSCLC	8	Assessment of whether [18F]-HX4 PET can predict patient outcome of radiotherapy. (#)	Nottingham University Hospitals NHS trust (NUH, UK)	N/A	NCT02976883
**IMCISION**	Ib II	Monocentric (EU)	Active, and recruiting	HNSCC	32 *	Examination of feasibility and safety of checkpoint blockade in combination with SOC in HNSCC and its potential impact on tumor hypoxia as measured by [18F]-HX4 PET. (#)	Netherlands Cancer Institute (NKI-AVL, NL)	N/A	NCT03003637

N/A: not available; NSCLC: non-small cell lung cancer; HNSCC: head and neck squamous cell carcinoma; GBM: glioblastoma multiforme; SOC: standard of care; p.i.: post-injection. Patient numbers are based on actual enrollment of patients found in the associated publication; * indicates the estimated patient enrollment of still active clinical trials; # indicates that no results have been posted yet.
